# Contemporary Trends in Same-Day Versus Deferred Discharge After Left Atrial Appendage Occlusion

**DOI:** 10.1016/j.jacadv.2023.100261

**Published:** 2023-03-08

**Authors:** Alejandra Chavez Ponce, Ahmed El Shaer, Samian Sulaiman, Alyssa Harris, Samuel F. Hohmann, Trevor Simard, Andrew M. Goldsweig, Mohamad Alkhouli

There is a growing interest in same-day discharge (SDD) after structural heart interventions in current practice. However, contemporary data on SDD after left atrial appendage occlusion (LAAO) are limited to single center feasibility reports.^1^^,^^2^ We sought to assess adoption rates and potential cost reduction associated with SDD after LAAO in the United States.

We utilized a large academic consortium database (Vizient Clinical Database) to identify patients who underwent LAAO between January 1, 2016, and December 31, 2021, using International Classification of Diseases (ICD)-10 procedural codes. The Vizient Clinical Database has been used to study practice patterns and outcomes of various transcatheter interventions including LAAO.^3^, ^4^, ^5^ We calculated the rate of SDD per calendar year. We also compared baseline characteristics, in-hospital outcomes, and cost between patients with SDD vs those discharged later (deferred discharge [DD] group). Categorical variables were compared using a chi-squared test, and continuous parameters were compared using the Student’s *t*-test and Wilcoxon rank test to compare the costs. Cost data were adjusted for inflation with year 2021 used as the reference year. The study was exempted by the Institutional Board Review at Mayo Clinic because it utilized de-identified publicly available data.

**Figure 1:**
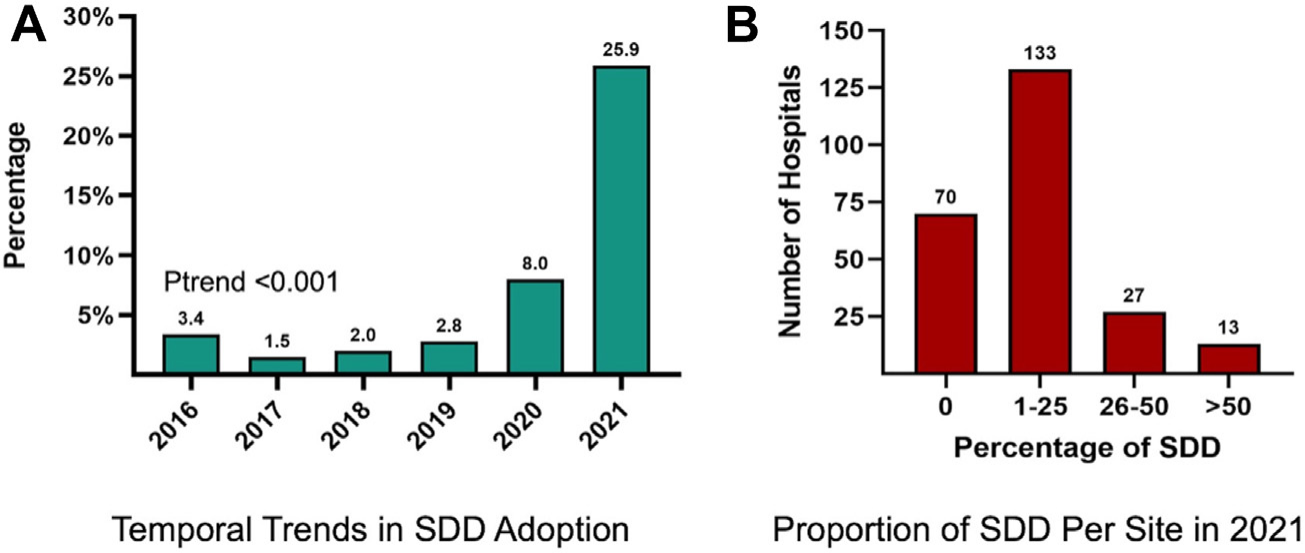
**Same-Day Discharge After Left Atrial Appendage Occlusion** **(A)** Temporal trend in the adoption of same day vs deferred discharge after LAAO. **(B)** Proportion of SDD to all LAAO cases at the 243 Participating Hospitals in 2021. LAAO = left atrial appendage occlusion; SDD = same-day discharge; Data from the Vizient Clinical Data Base used with permission of Vizient, Inc. All rights reserved.

A total of 45,135 patients were included in the analysis, of whom 5,083 (11.3%) were discharged on the same day. SSD accounted for <4% of cases prior to 2020 but increased substantially after (8% in 2020 and 25.9% in 2021) (*P* trend < 0.001) (Figure 1A). Regional variations were observed in SDD rates which were highest at hospitals in the Midwest (14.7%) and lowest at hospitals in the Northeast (5.5%) (*P* < 0.001). In 2021, the SDD rate was >50% at 13/243 hospitals (5.3%), between 25% and 50% at 29/243 hospitals (11.9%) and <25% at 201/243 hospitals (82.7%). Seventy hospitals (31.7%) did not discharge any patient on the same day after LAAO (Figure 1B).

Compared with patients in the DD group, those in the SDD group were younger (75.9 ± 7.8 vs 76.3 ± 8.1), and more likely to be male (62.3% vs 59.1%), and White (92.2% vs 88.1%). They also had fewer comorbidities: hypertension (84.6% vs 86.5%), coronary artery disease (45.4% vs 48.2%), peripheral vascular disease (6.8% vs 8.2%), congestive heart failure (32.7% vs 36.8%), chronic kidney disease (13.1% vs 22.6%), and chronic obstructive lung disease (19.1% vs 22.6%) (*P* < 0.001 for all). Utilization of intracardiac echo was low and comparable in SDD vs DD groups (3.0% vs 3.2%, *P* = 0.33). Major adverse events were higher in the DD group (4.3% vs 1.5%, *P* < 0.001), driven by more pericardial effusion (1.0 vs 0.1%), major bleeding (2.8% vs 1.1%), and major vascular complications (0.8% vs 0.2%), (*P* < 0.001 for all). Discharge to a facility (nonhome discharge) was more common in the DD group than in the SDD group (2.6% vs 0.5%, *P* < 0.001). Median cost of the LAAO hospitalization was significantly lower in the SDD vs DD groups ($26,649 [IQR: $20,958-$31,519] vs $28,015.2 [IQR: $19,554-$33,713], *P* < 0.001).

Our study suggests a substantial growth in the utilization of SDD after LAAO since 2020, albeit with considerable variations across sites. It also documents considerable differences in baseline characteristics, complication rates, and cost of the hospitalization between patients discharged same day vs those discharged later. This analysis is hindered by the limited granular information on the LAAO procedure in this database (eg, device manufacturer, type, and size used, residual leak, etc.), the lack of information on the reason for SDD vs DD, and the lack of long-term data beyond hospital discharge. Despite these limitations, this focused analysis represents the largest descriptive data on the contemporary trends in SDD after LAAO to date. Prospective studies are needed to assess the efficacy of SDD after LAAO with regards to readmission rates and device-specific outcomes.
